# NOD-like receptors: major players (and targets) in the interface between innate immunity and cancer

**DOI:** 10.1042/BSR20181709

**Published:** 2019-04-09

**Authors:** Fernando J. Velloso, Marina Trombetta-Lima, Valesca Anschau, Mari C. Sogayar, Ricardo G. Correa

**Affiliations:** 1 Department of Pharmacology, Physiology and Neuroscience, Rutgers-New Jersey Medical School Newark, NJ, U.S.A.; 2 Cell and Molecular Therapy Center (NUCEL), Internal Medicine Department, School of Medicine, University of São Paulo (USP), São Paulo, SP, Brazil; 3 Institute for Integrative Systems Biology (I2SysBio), Universitat de Valencia-CSIC, Valencia 46980, Spain; 4 Biochemistry Department, Chemistry Institute, University of São Paulo, São Paulo, Brazil; 5 NCI-Designated Cancer Center, Sanford Burnham Prebys Medical Discovery Institute, La Jolla, CA, U.S.A.

**Keywords:** cancer, inflammasome, NLR, NOD1, NOD2, NF-kB

## Abstract

Innate immunity comprises several inflammation-related modulatory pathways which receive signals from an array of membrane-bound and cytoplasmic pattern recognition receptors (PRRs). The NLRs (NACHT (NAIP (neuronal apoptosis inhibitor protein), C2TA (MHC class 2 transcription activator), HET-E (incompatibility locus protein from Podospora anserina) and TP1 (telomerase-associated protein) and Leucine-Rich Repeat (LRR) domain containing proteins) relate to a large family of cytosolic innate receptors, involved in detection of intracellular pathogens and endogenous byproducts of tissue injury. These receptors may recognize pathogen-associated molecular patterns (PAMPs) and/or danger-associated molecular patterns (DAMPs), activating host responses against pathogen infection and cellular stress. NLR-driven downstream signals trigger a number of signaling circuitries, which may either initiate the formation of inflammasomes and/or activate nuclear factor κB (NF-κB), stress kinases, interferon response factors (IRFs), inflammatory caspases and autophagy. Disruption of those signals may lead to a number of pro-inflammatory conditions, eventually promoting the onset of human malignancies. In this review, we describe the structures and functions of the most well-defined NLR proteins and highlight their association and biological impact on a diverse number of cancers.

## NOD-like receptors

The innate immune system is our first line of defense against infections from an enormous diversity of microbes and viruses. The human innate immunity relies on a wide range of receptors and complex downstream networks which respond against infectious pathogens. Activation of these immune pathways leads to a broad range of pro- and/or anti-inflammatory signals, including the secretion of interferons, tumor necrosis factors and cytokines [1]. Disruption in the balance of these signals may lead to chronic inflammatory states and directly affect cellular processes, such as cell cycle progression and apoptosis, creating a background context for the rise of maladies, such as cancer [1,2].

In humans, innate immune receptors are classified into several families [3]. Amongst the most well-characterized receptors are the TLRs (Toll-like Receptors) and NOD-like receptors (NLRs) [NACHT (NAIP (neuronal apoptosis inhibitor protein), C2TA (MHC class 2 transcription activator), HET-E (incompatibility locus protein from Podospora anserina) and TP1 (telomerase-associated protein), and Leucine-Rich Repeat (LRR) domain containing proteins] [4–6]. While TLRs act as surface receptors found in cell and organelle (endosome) membranes, the NLRs are cytosolic receptors involved in the detection of intracellular pathogens and endogenous byproducts of tissue injury [7]. The NLRs are also known as a subgroup of pattern recognition receptors (PRRs), which act as innate immunity ‘sensors’ of pathogen-associated molecular patterns (PAMPs) and danger-associated molecular patterns (DAMPs) [8,9]. Typically, the PAMPs recognized by NLRs are bacterial cell-wall derivates [10], microbial toxins [11], viruses [12] or even whole pathogenic microbial organisms [13]. The DAMPs are host-derived molecules released by injured cells, including extracellular ATP [14], hyaluronan [15] and monosodium urate (MSU) [16]. Therefore, NLRs act as key activators of innate immune responses which, upon detection of cell damage and infections, may lead to the expression and/or activation of stress kinases, interferon response factors (IRFs) and inflammatory caspases [17–21].

### NLR protein structure and subfamilies

**Figure 1:**
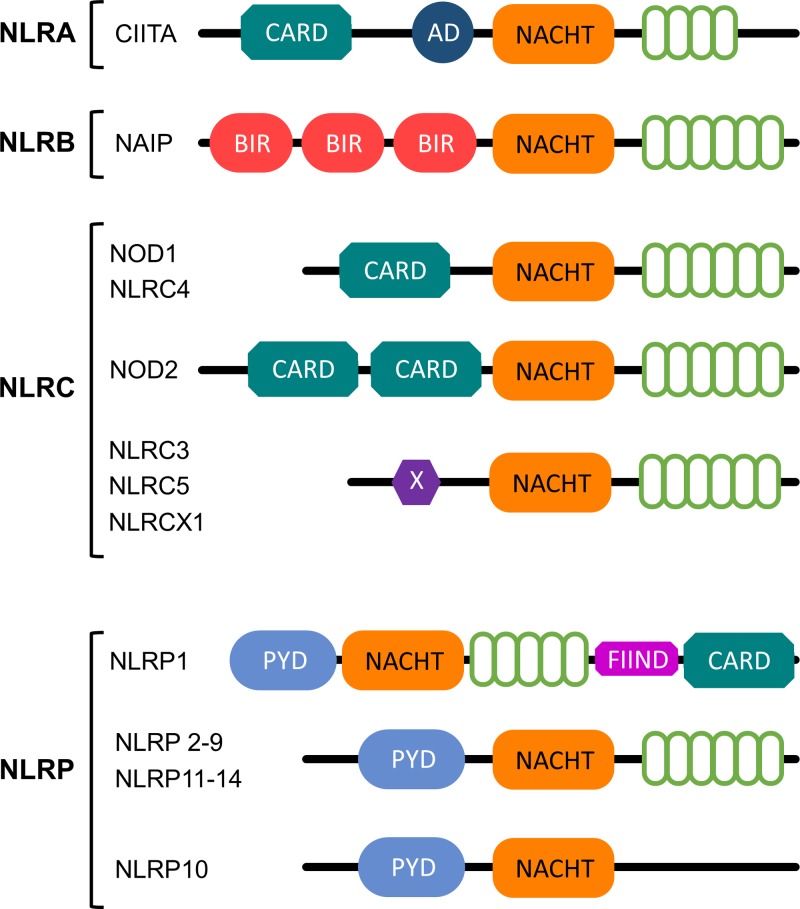
Protein structure representation of each NLR subfamily Respective domains are indicated as follows: **CARD:** Caspase recruitment domain; **AD**: Acidic transactivation domain; **NACHT**: NAIP (neuronal apoptosis inhibitor protein), C2TA (MHC class 2 transcription activator), HET-E (incompatibility locus protein from *Podospora anserina*) and TP1 (telomerase-associated protein); **BIR**: Baculoviral inhibitory repeat-like domain; **X:** Unknown;** PYD**: Pyrin domain. Green open circles represent **LRR** (Leucine-rich repeat).

A number of NLR homologs have been described in both vertebrate and invertebrate species [22]. In humans, the NLR protein family comprises 22 members [23–25]. All NLR proteins share a typical architecture, including: (i) a centrally located nucleotide-binding NACHT domain, which mediates self-oligomerization and is essential for ATP-dependent NLR activation; (ii) an N-terminal effector domain, which interacts with adaptor molecules and downstream effectors to mediate signal transduction; and (iii) a C-terminal region, comprising variable numbers of LRR domains, involved in the recognition of molecular patterns (Figure 1) [4]. Specifically, human NLRs are divided into four subfamilies, according to the nature of their N-terminal regions. These regions may contain (i) an acidic transactivation domain (AD) (NLRA subfamily), (ii) a baculoviral inhibitory repeat-like domain (BIR) (NLRB subfamily), (iii) a caspase activation and recruitment domain (CARD) (NLRC subfamily) or (iv) a pyrin domain (PYD) (NLRP subfamily) (Figure 1) [1,4,8,17,21,24].

**Figure 2:**
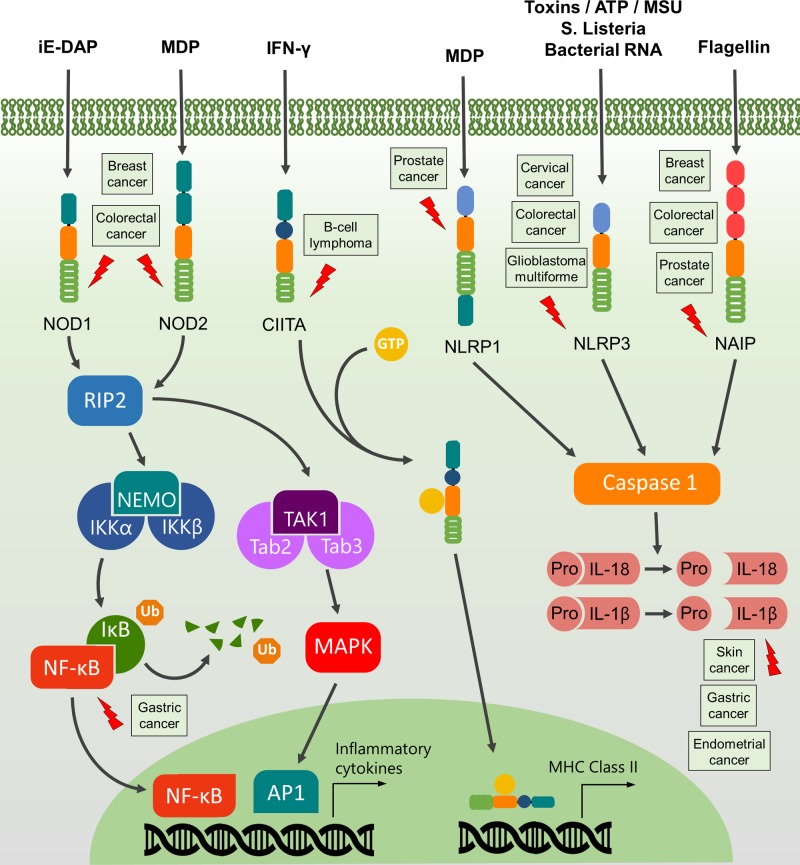
NLR signaling pathways (related to prototypical members of each subfamily) and their correlation with cancer Lightning arrows indicate specific signaling nodes or receptor(s) for which gene mutations or alterations in expression levels have been reported in association with major types of cancer (adjacent boxes).

The NLRA subfamily comprises a sole member, namely: the Class II Major Histocompatibility Complex Transactivator (CIITA). Apart from the AD domain, CIITA displays four LRRs and a GTP binding domain (Figure 1). GTP binding facilitates the protein transport into the nucleus, where it acts as a positive regulator of class II major histocompatibility complex gene transcription (Figure 2) [26]. In this case, transcriptional activation is not achieved through DNA binding, but via an intrinsic acetyltransferase (AT) activity [27,28]. Similarly, the NLRB subfamily comprises only one member, namely, the NLR Family Apoptosis Inhibitory Protein (NAIP). NAIP is an anti-apoptotic protein which acts by inhibiting (i) the activities of Caspase (CASP) 3 (CASP3), CASP7 and CASP9 [29], (ii) the autocleavage of pro-CASP9 and (iii) the cleavage of pro-CASP3 by CASP9 [30]. NAIP is a mediator of neuronal survival in several pathological conditions, preventing apoptosis induced by a variety of signals [31].

NLRC is the second largest subfamily of NLRs, consisting of six members: nucleotide oligomerization domain 1 (NOD1) (NLRC1), nucleotide oligomerization domain 2 (NOD2) (NLRC2), NLRC3, NLRC4, NLRC5, and NLRX1. The NLRC3, NLRC5 and NLRX1 members are classified in the NLRC subfamily due to their homology and phylogenetic relationships, although their N-terminal domains have not been fully characterized yet [32]. NOD1 and NOD2 (Nucleotide-Binding Oligomerization Domain-Containing Proteins 1 and 2) are considered as the founding NLRs as well as the two major members of the NLRC subfamily [33]. NOD1 and NOD2 recognize intracellular bacterial components, which enter the cell either via direct bacterial invasion or by other cellular uptake mechanisms [34,35]. NOD1 and NOD2 contain, respectively, one and two N-terminal oligomerization CARD domains and detect distinct motifs of peptidoglycans (PGNs) [36,37]. NOD1 recognizes d-γ-glutamyl-meso-DAP (l-Ala-γ-d-Glu-meso-diaminopimelic acid) (iE-DAP (d-γ-glutamyl-meso-DAP)) dipeptides, which are found in PGNs from all Gram-negative and some Gram-positive bacteria, while NOD2 recognizes the muramyl dipeptide (MDP) structure found in almost all bacterial types [33,36–38]. Therefore, NOD2 acts as a broader sensor of bacterial infection, while NOD1 recognizes a more specific subset of bacterial strains.

The NLRP subfamily of receptors consist of 14 members, characterized by the presence of an N-terminal pyrin (PYD) effector domain [39] which possesses a conserved sequence motif found in more than 20 human proteins, with functions in apoptotic and inflammatory signaling [8,39]. Within this subfamily, at least six receptors (NLRP1, NLRP3, NLRP6, NLRP7, NLRP12, NLRC4) have been reported to operate through formation of inflammasome complexes [39]. These NLRPs recognize various ligands originated from microbial pathogens (PGN, flagellin, viral RNA, fungal hyphae etc.), host cells (cholesterol crystals, uric acid etc.), and environmental sources (alum, asbestos, silica, alloy particles, UV radiation, skin irritants etc.) [8]. Studies have shown that *NLRP* genes play important roles in both the innate immune system and mammalian reproduction [8,40], suggesting that NLRPs might play a role in oogenesis and early preimplantation embryogenesis [8,40].

## NLR signaling and inflammasome-related pathways

NLR activation is translated through distinct subpathways to achieve pro- or anti-inflammatory responses (Figure 2). The downstream signals involved are modulated by the type of ligands bound to the NLR and may also depend on the cellular context. For instance, NOD1 and NOD2 receptors bind to the membrane of early endosomes in the cytoplasm, specifically interacting with the actin cytoskeleton in order to maintain an inactive state [41,42]. PGNs that are transported through the membrane [43–48] are promptly recognized by these NOD receptors. Ligand-bound NOD1 and NOD2 self-oligomerize through CARD–CARD interactions, using the endosomal membrane as a scaffold for the assembly of signaling complexes [46,49]. Oligomerized NODs send signals via the serine/threonine receptor-interacting protein 2 (RIP2) kinase [50], which, in turn, mediates ubiquitination of the nuclear factor κB (NF-κB) essential modulator (NEMO)/IKKγ complex and, consequently, activation of NF-κB and production of inflammatory cytokines [4,51,52]. Furthermore, poly-ubiquitinated RIP2 also recruits TAB (TGF-β-activated kinase 1 and MAP3K7-binding protein) and its associated kinase TAK1 (TGF-β-activated kinase 1) [53,54]. TAK1 is a downstream activator of stress kinase, mitogen-activated protein kinase (MAPK) cascades, which activate JNK (c-Jun N-terminal kinase) and p38 MAPK toward activator protein 1 (AP-1) transcriptional activity [38,55,56].

Both NOD1 and NOD2 also activate the host response through an alternate pathway, independent of RIPK2 and NF-κB signaling [21]. These receptors can detect intracellular bacteria which cross the plasma membrane and then recruit the autophagic essential adapter protein ATG16L1 to the bacterial entry site, promoting highly specific lysosome-mediated degradation of the invading microbe by the autophagic machinery [21,57–59]. In addition to detecting bacterial components, NOD1 and NOD2 receptors also monitor the cytoplasmic environment, responding to cytoskeleton perturbations and ER stress [60,61], which, ultimately, activate autophagy [62–64] and NF-κB-driven inflammation [61,65,66]. This effect allows these NOD receptors to respond to pathogens that do not produce specific PGNs [67].

### Inflammasome-forming NLRs

The inflammasome is a multiprotein intracellular complex, which is frequently formed in response to several pathophysiological stimuli [67]. Despite its cytosolic localization, inflammasome structures are capable of launching an effective immune response against bacteria, fungi and viruses [68]. Indeed, inflammasome activation is an essential component of the innate response, playing a critical role in clearance of pathogenic insults and/or damaged cells [69].

In brief, the inflammasome structure includes: a sensor (NLR), an adaptor protein (ASC (apoptosis-associated speck-like protein containing CARD)) and an effector molecule (pro- CASP1) [70]. ASC is a bipartite protein consisting of a PYD and a caspase recruitment domain (CARD) [39,71]. In resting cells, caspase-1 is present in a catalytically inactive pro-form (zymogen) called pro-caspase-1 [72]. Caspases have long been established as executioners of the apoptotic response, also contributing to inflammasome activation [69].

The inflammasome activation initiates through the auto-coactivation of caspase-1, resulting in cleavage of pro-interleukin-1β (pro-IL-1β) and pro-interleukin-18 (pro-IL-18) [73,74] into their mature and active forms (IL-1β and IL-18, respectively). The secretion of these cytokines may lead to pyroptosis, a term used to describe the inherently inflammatory process of CASP1-dependent programmed cell death [75,76]. Inflammasome-independent sources of IL-1β have also been suggested to contribute to inflammatory disease pathogenesis; however, very little is known about the molecular regulation of these pathogenic pathways [77].

As previously indicated, several NLRs play a role in the formation of inflammasomes, namely: NLRP1, NLRP3 and NLRC4 [70]. Other less characterized inflammasome structures include NLRP2, NLRP6, NLRP7, NLRP12, as well as AIM2-like receptor (ALR) proteins [78]. Interferon γ-inducible protein 16 (IFL16) has also been suggested to assemble inflammasomes and induce caspase-1 activation in macrophages, indicating differential functions of IFL16, depending on the type of cell infected [79].

NLRP1 was the first NLR family member reported to form an inflammasome complex [80]. NLRP1 has been described to bind directly to its ligand MDP *in vitro*, with this interaction apparently being sufficient to activate the inflammasome assembly [70]. Genetic variation in the human *Nlrp1* gene has been linked to increased susceptibility to certain autoimmune diseases [81], systemic lupus [82] and cancer [179]. Studies have also demonstrated a genetic association of polymorphisms in *Nlrp1* gene in driving the tumorigenic process, which leads to an increase in the production of downstream mediators (i.e. CASP1 and IL-1β) in malignant melanoma [83].

*NLRP1*-like genes are found in most, if not all, mammalian species for which a genome has been sequenced, including primates, rodents, ungulates and marsupials [84]. Humans express only one *NLRP1* gene, while the mouse genome contains three *Nlrp1* paralogs named *Nlrp1*a, *Nlrp1*b and *Nlrp1*c [79]. Nlrp1a and Nlrp1b contain all domains characteristic of murine NLRs, contrary to the Nlrp1c protein which is truncated so they lack the CARD domains [85]. The murine NLRP1b is involved in the mechanism by which *Bacillus anthracis* infection activates caspase-1 [86]. NLRP1b also serves as an inflammasome sensor for *Toxoplasma gondii*, leading to an inflammasome response in rats and, consequently, limiting parasite load and dissemination [87]. Still, more studies are warranted to describe the precise mechanism of *T. gondii* recognition by NLRP1b.

To date, NLRP3 (also known as cryopyrin and NALP3) is the most fully characterized member of the NLRs family [88]. The NLRP3 inflammasome is activated by a number of factors, which include: Gram-positive bacteria, viruses (such as influenza), fungi and protozoa, toxins (such as hemolysin), ATP, potassium efflux, and reactive oxygen species (ROS) [70,89–91]. In addition to the microbial and endogenous activators mentioned above, RNA and mitochondrial DNA have also been described as NLRP3 activators [92]. NRLP3 lacks a CARD, therefore, cannot recruit procaspase-1 without the presence of the adaptor molecule ASC [73]. NLRP3 interacts with ASC via PYD homophilic interactions [73]. Some studies link various adaptor proteins, such as guanylate-binding protein (GBP) [93,94], thioredoxin (TRX)-interacting protein (TXNIP) [89], amongst others shown to be critical for mammalian host defense. Altogether, the NLRP3 inflammasome integrates multiple signals to protect the host against different forms of cellular stress [95]. Nevertheless, the mechanisms governing the formation and activation of the NLRP3 inflammasomes, in certain cellular contexts, still deserve further investigation.

NLRC4 is also an important sensor for the activation of caspase-1, particularly in macrophages infected with *Salmonella* strains [96]. This sensor is typically activated by a more streamlined set of ligands, which includes bacterial flagellin and components of the bacterial T3SS (Type 3 secretion system proteins) [97]. NLRC4 appears to detect these ligands by recognizing pathogen derivatives, which are secreted into the host cell cytosol by certain bacterial strains [84].

## Impact of NLRs on cancer

### Chronic inflammation and cancer onset

Inflammation has a dual role in cancer onset and progression. Pro-inflammatory condition has been described as a crucial state for cancer onset, progression, angiogenesis, and metastasis [98–100], being related to chronic low-grade activation of the immune system as a result of the production of several downstream pro-inflammatory factors [101]. On the other hand, immunosurveillance can prevent cancer onset and limit tumor growth [102,103].

Biomolecules that are produced by tumor-infiltrating immune cells, such as cytokines, proteases, reactive oxygen and nitrogen species, can influence the microenvironment and act as intermediates in these pathological processes [104–106]. The microenvironmental changes caused by the immune infiltrate include alterations (i) in the tumoral extracellular matrix and (ii) in the interaction between the different cell populations of the tissue, resulting in epigenetic modifications, epithelial–mesenchymal transition (EMT), oncogenes expression promotion and silencing of tumor suppressors [107–110]. Collectively, these alterations may orchestrate cell growth and survival, migration and/or angiogenesis, therefore promoting tumor progression and metastasis [111–113].

One of the most prominent cascades involved in tumor promotion is NF-κB, a key pathway in innate immunity and inflammation, which frequently appears as an interesting therapeutic target [114,115]. Directly linked to NF-κB, inflammasomes and their effector proteins are associated with different chronic pro-inflammatory conditions, and can either promote tumorigenesis or act as key players in immunosurveillance [116,117]. Interestingly, NF-κB also exerts a critical regulatory role during development. Manipulation of NF-κB members in a diverse range of animal models results in severe developmental defects during embryogenesis, very often leading to embryonic lethality [118]. For instance, inactivation of the NF-κB pathway in chicks induces functional alterations of the apical ectodermal ridge, which mediates limb outgrowth [119,120]. In mice, the absence of NF-κB activity leads to prenatal death due to defects in organogenesis and endoderm progression [121,122]. One major protein complex of this pathway, known as IκB kinase (IKK (inhibitor of nuclear factor κB kinase)), directly regulates NF-κB activation also during development of early vertebrates [123]. The IKK complex is mainly composed by two catalytic subunits (IKK1 and IKK2) and one scaffolding molecule (NEMO). IKK2 is the major cytokine-responsive IκB kinase [124,125] and, contrarily, IKK1 seems to be a repressor of NF-κB activity in certain biological and cell-specific conditions [123]. For instance, *Ikk1* knockdown in zebrafish embryos leads to head-to-tail malformations due to up-regulation of NF-κB-responsive genes and NF-κB-dependent apoptosis [123]. Conversely, *ikk1* overexpression leads to midline structure defects (*no tail*-like phenotype) associated with the repression of NF-κB activity [126]. Mechanistically, Ikk1 seems to sequester the non-catalytic subunit NEMO from active IKK complexes, therefore blocking NF-κB activation. Indeed, truncation of the NEMO-binding domain (NBD) in Ikk1, as well as increased availability of NEMO *in vivo*, is able to rescue the *Ikk1* overexpression phenotype [123]. Altogether, the significance of NF-κB during early development certainly justifies the biological impact of this pathway in the onset and progression of various proliferative diseases, including cancer.

Here, we briefly discuss the inflammation mechanisms driven by distinct NLRs and their association with a substantial number of relevant malignancies. A snapshot of major cancer types associated with each NLR is shown in Figure 2, while a list of reports linking NLRs to a number of cancers is presented in Tables 1 and 2.

**Table 1:** Summary of reported associations between members of NLRs subfamilies A, B, and C, and cancer progression

NRL subfamily	Member	Associated cancer	Associated phenotype	Molecular mechanisms	References
NLRA	CIITA	Primary mediastinal B-cell lymphoma	Tumoral immune evasion	Decrease in surface MHC II and increase in CD274/PDL1 and CD273/PDL2	[128,129]
NLRB	NAIP	Breast	Higher expression in tumor samples	–	[131]
		Colorectal	Lower expression in tumor samples; depleted mice are more susceptible to colitis-associated cancer	Increase in STAT3 expression and failure to activate p53	[134,135]
		Prostate	Higher expression in advanced prostate cancer submitted to androgen deprivation therapy; possible contribution to docetaxel resistance	Expression is induced by NF-κB	[138]
NLRC	NOD1	Breast	SNPs associates with a higher cancer risk; inhibits ER-dependent tumor growth; deficiency correlates with tumor growth, an increased sensitivity to estrogen-induced cell proliferation, and impaired Nod1-dependent apoptosis; reduced cell proliferation and increased clonogenic potential *in vitro*	Apoptosis mediated by caspase 8 in a RIP2-dependent mechanism	[142–146]
		Colorectal	Expression in T cells is associated with reduced susceptibility to chemically induced colitis and tumorigenesis; limits inflammation and its induced tumorigenesis	Reduction of inflammation induced tumorigenesis in an IFNγ-mediated mechanism	[150]
		Gastric	SNPs associated with *Helicobacter pylori* infection and gastric lesions; up-regulated upon *H. pylori* infection and associates with a higher inflammatory state in GC	Activation of TRAF3 and suppression of Cdx2	[160–162]
	NOD2	Breast	SNPs associated with a higher cancer risk; reduced cell proliferation and increased clonogenic potential *in vitro*	–	[142,143,146]
		Colorectal	Deficient expression associates with higher susceptibility to experimental models of CRC and induced instability in the composition of gut bacteria; limits inflammation and ts induced tumorigenesis	Inhibition of NF-κB and MAPK pathways through the induction of IRF4	[156,157]
		Gastric	SNPs associated with *H. pylori* infection and gastric lesions	–	[159]
	NLRC3	Colorectal	Reduced expression correlated with cancer progression; suppression of cellular proliferation and induction of cell death	Inhibition of the PI3K-mTOR signaling pathway through interaction with PI3K, TRAF6, and mTOR, supression of c-Myc activity, FoxO3a and FoxO1	[148,151]
	NLRC4	Breast	Poor prognosis	Upon obesity, expression in myeloid cells leads to IL-1β expression and VEGFA-dependent angiogenesis	[141]
		Colorectal	Reduced expression correlates with cancer progression; mediates higher proliferantion and apoptosis evasion during tumorigeneis in casp-1 deficient mice	–	[148]
	NLRC5	Colorectal	Reduced expression correlates to impaired CD8^+^ T-cell activation and poor patient prognosis; higher cancer risk	Impaired MHC I pathway	[149,153–155]
		Gastric	Expression associated with lymph nodes and tumor node metastasis	–	[162]

Abbreviations: Cdx2, caudal-related homeobox 2; CRC, colorectal cancer; ER, estrogen receptor; GC, gastric cancer; PDL1, programmed death ligand 1; PDL2, programmed death ligand 2; VEGFA, vascular endothelial growth factor A.

**Table 2:** Summary of reported associations between NLRP and atypical genes and cancer progression

NRL subfamily	Member	Associated cancer	Associated phenotype	Molecular mechanisms	References
NLRP	NLRP1	Skin	Promotes migration, IL-1β processing, evasion of apoptosis and hyperplasia	IL-1β processing, Caspase-1 cleavage, inhibition of caspase-2, -3/7, and -9 activities	[209,210–212,214]
		Prostate	Up-regulated in experimental model of inflammation by formalin injection *in situ*	Increase in IL-1β, IL-18, and caspase-1 expressions	[205]
		Cervix	SNP associated with lower oncogenesis related to HPV infection	–	[166]
	NLRP3	Cervix	SNP associated with lower oncogenesis related to HPV infection, higly expressed in an inflammatory context upon LPS treatment	Caspase-1 cleavage, IL-1β expression and processing	[166,167]
		Colorectal	Expression in macrophages: promotes invasion, migration, metastasis of tumor cells	Expression in macrophages: leads to caspase-1 cleavage, NLRP3–ASC–caspase-1 complex formation, and IL-1β processing and secretion	[169,171,173–175]
			Expression in tumor cells: promotes EMT; depletion leads to higher tumor burden, liver metastasis, and impariment of NK cell maturation	Expression in tumor cells: promotes EMT in a caspase-1 independent mechanism through Snail1 expression; depletion leads to IL-18 impairment, and consequent IFN-γ and STAT1 inhibition	
		Gastric	SNPs associated with higher cancer risk; expression in macrophages was found to be associated with aggressiveness	IL-1β secretion	[190]
		Glioblastoma	Promotes EMT, higher migratory and invasive potential, proinflammatory signaling, IL-1 production, ionizing radiation (IR) treatment resistance, cellular senescence after IR, resistance to apoptosis	IL-1β processing, AKT/PTEN pathway and Stat3 activation	[197–200]
		Skin	Promotes migration, IL-1β processing and hyperplasia	IL-1β processing, Caspase-1 cleavage, NFKβ pathway	[211,212,214]
	NLRP6	Colorectal	Associated with self-renewal of the colon epithelium upon injury, integrity and homeostasis of the epithelial barrier, depletion leads to higher tumor burden	Down-regulation of the cytokine IL-22BP in an IL-18-dependent mechanism, promotes inflammation through CCL-5, IL-18 and IL-6 pathway activation	[152,179,180]
	NLRP7	Endometrial	Correlates with depth of tumor invasion	–	[187]
		Gastric	Deficiency associated with lymph node metastasis and poor overall survival	Senescence mediated by P21 and Cyclin D1	[191]
	NLRP12	Colorectal	Its depletion leads to higher tumor burden	Modulation of noncanical NF-κB through TRAF3 and NIF, AKT and ERK pathways	[181,182]
		Gastric	SNPs associated with higher cancer risk	–	[189]
Atypical	NWD1	Prostate	Expression correlates with tumor progression	Its expression is modulated by SRY	[219]
				Regulates PDEF expression	
				Its depletion reduces AR levels and androgen-responsive genes	

Abbreviations: AR, androgen receptor; HPV, human papillomavirus; IL-22BP, IL-22 binding protein; LPS, lipopolysaccharide; NK cell, natural killer cell; PDEF, prostate-derived Ets factor; SRY, sex-determining region Y.

### NLRA-associated cancers

#### B-cell lymphoma

B-cell lymphomas comprise approximately 85% of all non-Hodgkin’s lymphomas (NHL), amongst which the primary mediastinal large B-cell lymphoma (PMBCL), a subtype of diffuse large B-cell lymphoma (DLBCL), sums up approximately 10% of the cases [127]. The incidence of PMBCL is higher in young adults and adolescents, with a metastatic potential to invade surrounding tissues [127]. Analysis of the *CIITA* sequence in PMBCL patient samples revealed the presence of structural genomic rearrangements, missense, nonsense, and frameshift mutations in 53% of the clinical cases [128]. These alterations led to decreased CIITA protein levels and, consequently, suppression of MHCII on the cell surface [128]. A similar study described that genomic breaks in the *CIITA* locus were present in 38% of the PMBCL samples and 15% of classical Hodgkin lymphoma (cHL) [129]. These alterations in *CIITA* sequence are associated with the down-regulation of surface MHC II, and increased expression of ligands of the receptor molecule programmed cell death 1, programmed death ligand 1 (PDL1) and programmed death ligand 2 (PDL2) [129]. These data suggest that CIITA has an essential role in PMBCL progression [128,129].

### NLRB-associated cancers

#### Breast cancer

Breast cancer is the most prevalent cancer in women, accounting for 29% of all diagnosed cancers in females [130]. Little is known about NAIP’s role in breast cancer, but *NAIP* mRNA levels have been well detected in tumor samples, while no expression is observed in control tissues [131]. In addition, *NAIP* expression in these malignant tissues is correlated with tumor size, but not with relapse-free survival [131]. More mechanistic studies are still warranted to confirm whether NAIP is relevant to breast cancer biology.

#### Colorectal cancer

Colorectal cancer (CRC) has the third highest cancer incidence worldwide, accounting for 9% of all cases, and is the fourth cause of death by cancer [132,133]. NAIP might also play an important role in preventing CRC onset [134]. Not only *NAIP* expression in colon cancer samples was found to be lower than in normal mucosa [135] but also, based on a model of colitis-associated cancer, mice lacking NAIP paralogs (Naip1-6) display a higher susceptibility for CRC in an inflammation-independent mechanism [134]. Furthermore, these knockout mice displayed increased *STAT3* expression and failed to activate p53 upon carcinogen exposure [134]. This suggests that NAIPs may act as tumor suppressors *in vivo* by inducing apoptosis in carcinogen-affected cells.

#### Prostate cancer

Prostate cancer (PCa) is the most common cancer in men [136,137]. Advanced PCa, submitted to androgen deprivation therapy, displays increased *NAIP* expression, which may possibly contribute to docetaxel resistance [138]. One possible explanation is that androgens generally inhibit responsive elements in NF-κB transcription factors promoters, decreasing their expression [138,139]. Therefore, it was verified by chromatin immunoprecipitation (ChIP) that, upon hormonal deprivation, NF-κB largely interacts with κB-like sites along the *NAIP* locus to promote its transcription activation [138]. These data suggest that NAIP levels may correlate with drug resistance in the treatment of PCa, but further experiments are needed to explore the role of NAIP in these mechanisms.

### NLRC-associated cancers

#### Breast cancer

Obesity has been associated with a poor prognosis of breast cancer patients, since adipose cells stimulate angiogenesis and synthesize estrogen, a primary female hormone that impacts tumor growth and metastatic potential [140]. For instance, in an orthotopic model, obese mice displayed higher tumor-infiltrating myeloid cells content and higher tumor-angiogenesis [141]. Interestingly, myeloid cells from obese mice display increased *NLRC4* expression and, consequently, IL-1β production. Cross-talk between tumor tissue and immune infiltrates also leads to vascular endothelial growth factor A (VEGFA)-mediated angiogenesis in an NLRC4-dependent manner, therefore driving disease progression [141].

A number of *NOD1* and *NOD2* SNPs have been associated with a higher risk of cancer development in many malignancies [142,143]. Although no tumor suppressor activity has been described for NOD2, NOD1 seems to have important tumor suppressor activity in estrogen receptor (ER)-dependent breast cancer, using an SCID mice xenograft model [144]. In ER-positive MCF-7 cells, NOD1 deficiency correlates with tumor growth, an increased sensitivity to estrogen-induced cell proliferation and impaired Nod1-dependent apoptosis. Correspondingly, in the same cells, *NOD1* overexpression inhibited ER-dependent tumor growth and reduced estrogen proliferative response *in vitro* [144]. Apparently, Nod1-dependent apoptosis is mediated by a caspase 8-cascade in an RIP2-dependent manner [145]. More recently, it has been described that overexpression of either NOD1 or NOD2, in the triple negative Hs578T cells, is able to reduce cell proliferation but increase clonogenic potential *in vitro* [146]. The proteomic profile of these overexpressing cells suggests the involvement of several inflammation- and stress-related pathways (intersecting NF-κB, PI3K and MAPK cascades) in the modulation of protein degradation processes, cell cycle and cellular adhesion [147]. The disruption of these critical systems suggests a functional link between NOD1/NOD2 and the proliferation and migration of triple negative breast cancer cells [147]. Although NOD1 tumor suppressive role is evidenced in ER-dependent tumors [144], both NOD1 and NOD2 appear to be relevant for the aggressive potential of breast cancer *in vitro*.

#### CRC

The expression of certain NLRCs has also been found to be modulated in CRC [148–150]. A combined analysis of TCGA (http://cancergenome.nih.gov) and Oncomine (https://www.oncomine.org) datasets, with mRNA expression analysis of tissue samples, revealed that *NOD1* and *NOD2* expression is usually increased, while *NLRC3* and *NLRC4* expression is reduced in CRC [148]. Furthermore, TCGA data analysis revealed that *NLRC3* expression inversely correlates with the American Joint Committee CRC staging [148]. Based on this staging, CRC is classified from stage I to IV in which (i) stage I tumors have breached beyond the inner lining of the colon, (ii) stage II tumors invaded the muscular wall of the colon, (iii) stage III tumors have reached the lymph nodes and (iv) stage IV tumors have metastasized to other organs besides the lymph nodes [148]. This correlation might be explained by recent reports describing the link between NLRC3 and the concomitant suppression of cellular proliferation and induction of cell death through the inhibition of the PI3K-mTOR signaling pathway in different node points [151]. Interestingly, *NLRC3* knockout mice, treated with azoxymethane and dextran sodium sulfate (colitis-associated CRC model), display an increased *C-MYC* expression and FoxO3a and FoxO1 phosphorylation (effectors of the PI3K-AKT pathways) [151]. Likewise, caspase-1-deficient mice submitted to the same treatments show increased epithelial cell proliferation in early stages of oncogenesis, and apoptosis evasion in additional stages in an NLRC4-dependent manner [152].

NLRC deficiencies are also correlated to immunosurveillance escape-mediated tumor progression [149]. Gene mutations, polymorphisms, loss of copy numbers, and methylation of the MHC class I transactivator *NLCR5* have been associated with MHC I pathway disruption and a higher cancer risk [149]. It is interesting to note a correlation between reduced *NLCR5* expression and higher CRC risk, especially in mismatch repair-deficient tumors [153–155]. Moreover, it has been proposed that reduced *NLRC5* expression also correlates to impaired CD8^+^ T-cell activation and poor patient prognosis [149].

Furthermore, NOD1 expression in T cells has been associated with a reduced susceptibility to chemically induced colitis and subsequent tumorigenesis, by limiting inflammation-induced tumorigenesis in an IFNγ-dependent mechanism [150]. Similarly, *NOD2* deficient mice appear to be more susceptible to experimental models of CRC [156]. Both NOD1 and NOD2 can inhibit NF-κB and MAPK pathways through induction of IRF4 [156] and, apparently, have a role in the suppression of inflammation-induced tumorigenesis [156]. Furthermore, *NOD2* deficient mice are seemingly more prone to colitis and colitis-related cancer due to induced instability in the composition of gut microbiome [157]. This increased susceptibility to inflammation could be prevented by (i) microbiota transplantation, (ii) antibiotics or (iii) anti-IL-6 neutralizing antibody treatment [157]. These findings reiterate the notion that NLRCs also influence tissue microenvironment and suppress CRC tumorigenesis.

#### Gastric cancer

*Helicobacter pylori* infection is a strong risk factor for gastric cancer (GC) [158]. *NOD1-* and *NOD2*-specific SNPs have been associated with *H. pylori* infection and gastric lesions [159]. In this context, expression of the epithelial-specific transcription factor *CDX2* is known to contribute to intestinal metaplasia (an event that precedes GC) and to be induced by *H. pylori* infection [160]. The NF-κB pathway has been implicated in induction of *CDX2* expression [160]. In contrast, NOD1-dependent activation of TRAF3, a negative regulator of NF-κB, may suppress *CDX2* expression [160]. This is somewhat contradictory to the findings in which, upon *H. pylori* infection, NOD1 is up-regulated and associated with a higher inflammatory state in GC [161].

*NLRC5* expression has been correlated with lymph nodes and tumor node metastasis in GC [162]. As a result, NLRC5 has been considered as an independent risk factor for the prognosis of GC patients [162]. The orchestrated expression of *NLRC5*, as well as of other NLRC proteins, may play an important role in GC onset, but more detailed studies are needed to better dissect their actual contribution to GC.

### NLRP-associated cancers

Amongst the NLRP subfamily members, the role of NLRP3 in cancer is the most well characterized (extensively revised in [163]). Here, we describe some of the main findings linking NLRPs to different human malignancies.

#### Cervical cancer

Cervical cancer is the second most common cancer type in women [164]. It has been found that persistent Human Papillomavirus (HPV) infection, associated with chronic inflammation, may lead to cancer onset [165]. Particularly, polymorphisms in *NLRP1, NLRP3* and *IL-18* have been associated with a lower HPV persistence and associated oncogenesis [166]. Using an inflammation model, human cervical cancer cells, positive for HPV-16 and treated with lipopolysaccharide (LPS), have indeed displayed increased levels of NLRP3, IL-1β, processed IL-1β, and cleaved caspase-1 [167].

#### CRC

Inflammation is highly associated with the onset of CRC. Inflammatory bowel disease (IBD), which comprises diseases such as ulcerative colitis and Crohn’s disease, is mainly a chronic inflammatory condition which is known to increase the overall risk of developing CRC by 4- to 20-fold [132]. NLRP3 has been proposed to be a link between IBD and CRC (reviewed in [168]). Interestingly, high-fat diet has also been associated with NLRP3 activation and increased tumor susceptibility [162,169]. High-fat diet leads to an increase in deoxycholic acid levels in the intestine, which, in turn, disrupts the cell monolayer integrity by decreasing the expression of the tight junction protein ZO-1 [170]. This disruption in the mucosal barrier leads to an increased tissue inflammation, mediated by NLRP3, and further polarization of M2 macrophages [162]. Likewise, azoxymethane-treated mice submitted to a cholesterol-rich diet show increased tissue inflammation and higher susceptibility to tumor development [169]. In fact, cholesterol inhibits the activity of AMPKα in macrophages, resulting in increased levels of mitochondrial ROS [169]. An oxidative microenvironment may then activate NLRP3, leading to (i) inflammasome formation, (ii) caspase-1 cleavage and (iii) IL-1β processing and secretion [169]. This cascade of events can be partially reverted by NLRP3 depletion [169].

*NLRP3* expression has also been found in macrophages infiltrated in CRC tissues, and the inhibition of NLRP3 pathway leads to decreased tumor cell migration, invasion and metastatic potential [171]. These data are supported by the evidence that treatment with a small-molecule AMPK activator (GL-V9), which acts as an anti-inflammatory molecule on macrophages, triggers autophagy and NLRP3 degradation, providing a protective effect against colitis and CRC [172].

Although *NLRP3* expression in tissue-infiltrated macrophages has been associated with higher susceptibility to CRC and its aggressiveness, its role in tumor cells is, at a first glance, controversial. NLRP3 has been found, for instance, to be highly expressed in the SW620 mesenchymal-like CRC cell line [173]. Moreover, HCT116 and HT29 epithelial-like CRC cell lines, when submitted to EMT through the treatment with TNF-α and TGF-β1, displayed an increase in *NLRP3* expression mediated by NF-κB [173]. In contrast, *NLRP3* or *CASP1* deficient mice are more susceptible to the CRC burden induced by azoxymethane-DSS-induced inflammation model [174]. This phenotype is associated with lower *IL-18* expression levels and, consequently, impairment of *IFN-γ* expression and suppression of STAT1 activation [174]. In addition, *NLRP3* knockout mice display augmented liver metastasis [175], which is also due to the impairment of IL-18 signaling. This suppression affects Fas ligand (*FasL*) expression in natural killer cells (NK cells), thus compromising their ability to kill FasL-sensitive tumor cells [175].

In accordance with the current data, *NLRP3* expression might be explored for the prevention of CRC. One example is its role as an effector of TRAIL (tumor necrosis factor related apoptosis-inducing ligand), an apoptosis-inducing protein whose use for cancer treatment has been currently evaluated [176–178]. In this context, mice submitted to the azoxymethane-DSS CRC model and treated with recombinant TRAIL displayed inhibition of macrophage recruitment to the damaged mucosa, therefore diminishing acute inflammation [176]. At the same time, TRAIL promoted tissue regeneration by NLRP3 activation, which induced IL-18 expression and promoted IL-1β secretion and caspase-1 cleavage [176]. These studies emphasize the multifunctional role of NLRP3, as well as the importance of the cross-talk between the different resident tissue cells and the CRC outcome.

Other members of the NLRP subfamily have also been related to CRC biology. For instance, NLRP6, typically produced by the stem-cell niche, acts on the self-renewal of the colon epithelium upon injury and, therefore, it is important for the integrity and homeostasis of the epithelial barrier [179]. Indeed, *NLRP6* deficient mice show impaired regeneration of the mucosa upon injury, and they are susceptible to colitis-associated tumor growth [179]. NLRP6 is involved in inflammation promotion by down-regulating the IL-22 binding protein (IL-22BP) which neutralizes IL-22 in an IL-18-dependent mechanism [180]. In addition, NLRP6 promotes inflammation through microbiota-induced activation of chemokine (C–C motif) ligand 5, IL-18 and IL-6 related pathways [69].

NLRP12 is another potential therapeutic target, since *NLRP12* knockout mice looks prone to colon inflammation and CRC, through enhanced activity of non-canonical NF-κB, ERK and AKT pathways, in both macrophages and tumor cells [181,182]. Nevertheless, due to the dual role of inflammation in cancer development, further studies are still warranted to better explore the clinical potential of some inflammasome-related proteins.

#### Endometrial cancer

The incidence rates of endometrial cancer have increased during last few decades and, nowadays, is considered the sixth most common cancer in women [183]. Its occurrence is associated with precursor hyperplasic lesions in more than 40% of cases [184]. Although IL-1 has been described to have an important role in endometriosis (a chronic inflammatory condition in which endometrial tissue grows outside the uterine cavity) [185,186], little is known about the inflammasome’s role in the development of this endometrial condition. The only available data so far refer to a statistical correlation observed between NLRP7 and the depth of the tumor invasion in the surrounding normal tissue [187], which is indeed promising but requires more detailed investigations.

#### GC

GC is the fourth most common type of cancer, and it is responsible for the second highest rate of cancer-related deaths [188]. Specific SNPs in some NLRP subfamily members, such as *NLRP3* and *NLRP12*, have been associated with increased risk of *H. pylori* infection (one of GCs most prominent risk factors) and also to GC itself [189]. *H. pylori*-challenged cells can lead to simultaneous down-regulation of *NLRP9* and *NLRP12* and up-regulation of the canonical NF-κB pathway [189]. Indeed, NLRP12 is a known inhibitor of the NF-κB pathway, and its inhibition might contribute to the maintenance of an active state of this signaling cascade [189].

*NLRP3* expression in macrophages has been found to be associated with GC aggressiveness [190]. In a physiological scenario, the microRNA miR-22 (expressed in the gastric mucosa) inhibits *NLRP3* expression and suppresses inflammation [190]. *H. pylori* infection suppresses miR-22, increasing *NLRP3* expression which, in turn, leads to IL-1β secretion and promotes the proliferation of epithelial cells and GC tumorigenesis [190]. Contrarily, it has been reported that *NLRP6* expression is reduced in ∼75% of the primary GC cases, and is associated with lymph node metastasis and poor overall survival [191]. *NLRP6* expression may suppress cancer cell proliferation by inducing senescence in a mechanism mediated by p21 and cyclin D1. In fact, overexpression of *NLRP6*, along with the inactivation of NF-κB and Mdm2, activates the p14ARF-p53 pathway and promotes senescence of GC cells [191]. This particular mechanism may be potentially explored for the GC treatment.

#### Glioblastoma multiforme

Glioblastoma multiforme (GBM), also known as Grade IV astrocytoma, is the most common type of brain tumors in adults, comprising approximately 17% of the cases [192,193]. GBMs are extremely aggressive tumors, displaying highly infiltrative growth patterns and a very poor prognosis, with a median overall survival of 15–18 months after diagnosis [192,194,195].

The tumor microenvironment plays a crucial role in GBM progression. In particular, the presence of activated microglial and macrophage cells are associated with higher aggressive phenotypes (reviewed in [196]). Amongst the soluble factors secreted by microglial cells, IL-1 is known to activate GBM cells, partially due to the activation of TGFβ pathway, and also to alter their secretome, resulting in the up-regulation of interleukin-8 (IL-8) and C–C motif chemokine ligand 2 (CCL2), and the down-regulation of collagen type IV α 2 chain (COL4A2) [197]. In human GBM cell lines, NLRP3 is also responsible by IL-1β processing [198]. IL-1 production in these cells leads to activation of the transcriptional factor Stat3, resulting in increased cellular migration and establishing a mesenchymal phenotype [198].

NLRP3 has been positively correlated to higher histological grades in astrocytomas [199]. *NLRP3* overexpression in human GBM cells promotes invasion, migration, proliferation, resistance to apoptosis and EMT via activation of the AKT pathway [199]. In addition, *NLRP3* expression has been linked to resistance against ionizing radiation therapy, leading to an increased number of senescent cells after this treatment [200]. Interestingly, this phenotype is partially reverted by NLRP3 inhibition [200]. Therefore, NLRP3 looks like a promising therapeutic target, and the use of NLRP3 inhibitors, such as β-Hydroxybutyrate or certain miRNAs, have been considered for GBM treatment [201,202].

#### PCa

Studies have shown that the presence of infiltrating immune cells in prostatic tissues is inversely correlated to PCa progression [203,204]. Prostatic inflammation, experimentally induced by intra-prostatic injection of formalin, leads to increased *NRLP1* expression and consequent increase in IL-1β, IL-18 and caspase-1 levels [205]. Highly metastatic PCa cells (DU145 and PC-3) secrete IL-18 binding protein (IL-18BP) after IFN-γ stimulation [206]. Coincidentally, IL-18BP levels in patient sera have been correlated with PCa aggressiveness [206]. This suggests that IL-18 neutralization might be a mechanism by which PCa cells bypass immunesurveillance and promote tumor development.

#### Skin cancer

Approximately 2–3 million skin cancers cases are diagnosed each year and their incidence has increased over the last decades [207]. Skin tumors can be classified as non-melanomas (derived from keratinized epithelial cells) or melanomas (derived from melanocytes) [190,191]. Melanoma accounts for 2% of the cases, being the most aggressive type of skin cancer, accounting for almost 10000 deaths per year [207,208].

Although inflammation may contribute to defense mechanisms against tumor onset, chronic skin inflammation can promote the development of benign and malignant lesions. For instance, using organotypic *ex vivo* skin models, treatment with IL-1 leads to an increase in epidermal thickness due to the proliferation of keratin-10- and involucrin-positive keratinocytes in the basal layer [209]. This higher proliferation rate is accompanied by an increased expression of the stress markers, S100 calcium binding proteins A8/9 (*S100A8/9*) and S100 calcium binding protein A7 (*S100A7*), known to be highly expressed in skin cancers, suggesting that inflammasome-dependent IL-1 production may be sufficient to induce skin hyperplasia [209].

The skin typically displays high expression levels of *NLRP1*, and gain-of-function mutations along this gene can lead to skin hyperplasia, including multiple self-healing palmoplantar carcinoma (MSPC) and familial keratosis lichenoides chronica (FKLC) [209]. *NLRP1* knockdown in metastatic melanoma cell lines induces lower caspase-1 activity and IL-1β production/secretion, but it also results in increased caspase-2, -3/7 and -9 activities, therefore promoting apoptosis [210]. Likewise, activation of NLRP1, but not of NLRP3, decreases caspase-2, -3/7, and -9 activities and consequent evasion from apoptosis [210].

Ultraviolet B (UVB) radiation is considered a major risk factor for skin cancer. Both NLRP1 and NLRP3 have been implicated in the first response to UVB in human keratinocytes [211,212]. UVB induces *NLRP1* and *NLRP3* expression, leading to inflammation onset through extensive IL-1β secretion [211,212]. Furthermore, specific SNPs in both *NLRP1* and *NLRP3* have been associated with susceptibility to nodular melanoma [213]. More recently, CRISPR inactivation of both NLRP genes revealed that *NLPR1* is, in fact, the main responsible for the cellular pro-inflammatory response against UVB radiation [214]. Nevertheless, a compound isolated from *Nigella sativa* seeds, called thymoquinone (2-isopropyl-5-methyl benzo-1,4-quinone), was found to inhibit migration of melanoma cells through inhibition of *NLRP3* expression and its related cascade, leading to a decrease in caspase-1 cleavage as well as IL-1 and IL-18 levels [215]. This suggests that both NLRP proteins may be relevant for the onset and progression of skin cancer.

### NLR-related proteins and cancer

#### PCa

Other cytosolic receptors, which are not fully categorized as NLRs but still share structural similarities, may also be of clinical relevance in the context of cancer development. For instance, NWD1 (NACHT and WD repeat domain-containing protein 1) is an NLR-related protein which carries a conserved NACHT domain and WD40 repeats instead of LRRs at the C-terminus [216]. Sequence homology analysis suggests this protein may be a novel NLR family member [216]. NWD1 also share homology with Apaf1 (Apoptotic peptidase activating factor 1), a cytoplasmic receptor that also possesses WD40 repeats instead of LRRs, and it is involved in caspase 9-mediated apoptosis [217,218]. It has been reported that *NWD1* expression elevates in the course of PCa progression. *In vitro* experiments demonstrated that sex-determining region Y (SRY) proteins may regulate the *NWD1* expression, which in turn regulate PDEF (prostate-derived Ets factor), a transcription factor which is known to bind and modulate the androgen receptor (AR). Furthermore, *NWD1* depletion reduces AR levels and androgen-responsive genes, suggesting a role for NWD1 in PCa via AR deregulation [219].

## Conclusion

Based on the data here described, we summarized how deregulation in the balance of NLR-related signals may lead to the onset of several types of cancer. Despite all the knowledge accumulated regarding these cytosolic receptors, the functional domains, ligand specificity and signal transduction events directed by each particular family member still remain to be better elucidated. At the same time, new atypical NLR members may continue to be uncovered, adding another layer of complexity to the studies involving innate immune sensors. A more in-depth understanding of how these receptors signal through different pathways, and how they interact to achieve a global impact in diverse pathologies, such as cancer, will be seminal to develop better diagnostic and prognostic tools, as well as more effective therapeutic strategies.

## Abbreviations

AD: acidic transactivation domain
AR: androgen receptor
ASC: apoptosis-associated speck-like protein containing CARD
AT: acetyltransferase
CARD: caspase recruitment domain
CASP1: caspase 1
CIITA: class II major histocompatibility complex transactivator
CRC: colorectal cancer
DAMP: danger-associated molecular pattern
EMT: epithelial–mesenchymal transition
ER: estrogen receptor
FasL: Fas ligand
GBM: glioblastoma multiforme
GC: gastric cancer
HPV: human papillomavirus
IKK: inhibitor of nuclear factor κB kinase
IRF: interferon response factor
IκB: inhibitor of nuclear factor κB
LRR: leucine-rich repeat
MAPK: mitogen-activated protein kinase
MDP: muramyl dipeptide
NAIP: NLR family apoptosis inhibitory protein
NACHT: NAIP (neuronal apoptosis inhibitor protein), C2TA (MHC class 2 transcription activator), HET-E (incompatibility locus protein from Podospora anserina) and TP1 (telomerase-associated protein)
NEMO: NF-κB essential modulator
NF-κB: nuclear factor κB
NLR: NACHT and LRR domain containing protein
NOD1: nucleotide oligomerization domain 1
NOD2: nucleotide oligomerization domain 2
NWD1: NACHT and WD repeat domain-containing protein 1
PAMP: pathogen-associated molecular pattern
PCa: prostate cancer
PGN: peptidoglycan
PMBCL: primary mediastinal large B-cell lymphoma
PRR: pattern recognition receptor
PYD: pyrin domain
RIP2: receptor-interacting protein 2
ROS: reactive oxygen species
TAK1: TGF-β-activated kinase 1
TLR: Toll-like receptor
TRAIL: tumor necrosis factor related apoptosis-inducing ligand
UVB: ultraviolet B
